# Supplementary material for: Network analysis of synthesizable materials discovery
Source: Nat Commun. 2019 May 1;10:2018. doi: 10.1038/s41467-019-10030-5 (PMC6494829; doi:10.1038/s41467-019-10030-5)
Supplement: Supplementary file 1 — Supplementary Information [file 41467_2019_10030_MOESM1_ESM.pdf]

Supplementary Information

Network analysis of synthesizable materials discovery

Aykol et al.

SUPPLEMENTARY METHODS

Subsampling to form the materials stability network: We extract a subset of tie-lines for the purposes of analyzing synthesizability in a computationally tractable way as necessary for the repeated network analysis required for the machine-learning stage (described later). Majority of the 40 million connections in the complete network are hetero-chemical, i.e. across materials with distinct chemistries that do not share a common element (e.g. NiAl-Li_2O). While such tie-lines are important for the complete picture of phase equilibria in the 90-dimensional chemical space, they likely do not play a primary role in driving stabilization and synthesis of materials. Stability of a material, i.e. whether a material is on the convex-hull, depends exclusively on those materials that share the same chemical space with it. For example, while $LiAlO_2$ can have thousands of tie-lines in the 89-D space, its stability is controlled only by those materials in the Li-Al-O chemical space. Therefore, the subsequent tie-lines $LiAlO_2$ forms with those materials are more relevant for synthesis than the rest of the network. With these principles, we build the materials stability network efficiently by:

- Determining the nodes (stable materials) of the network.
- Going over this set of all nodes and computing their connections (tie-lines) in their respective chemical spaces.
- Combining these tie-lines by removing duplicates and obtaining a unique set.

This materials stability network then includes only those tie-lines that play a role in determining stability of at least one material, yielding a network of $\sim$ 200,000 tie-lines.

Temporal evolution of the stability networks: Time dependence of the network can be obtained as the retrospective analysis of the present master network. Stable nodes are determined to be the ones in the present network. At a given past time, the network includes only the materials that were already discovered at that time, and only the tie-lines between those materials as defined in the master network. In other words, in this implementation, tie-line information is not reconstructed at a given past time, but the set of tie-lines from the present status of the network are simply added according to the time of discovery as described above. Other implementations are possible, but the present method avoids the computational cost of reconstructing the convex-hull, and is therefore the preferred method.

Computation of temporal-evolution of network properties: For each past and present network, multiple network properties for each node (i.e. a material in the network) are calculated as described in the manuscript. For a material already existing in a given network (i.e. materials that were already discovered at the time of the network), network properties are readily computable. For stable materials that do not exist in that network at that time (i.e. those that were not discovered then), point calculations of network properties are done. In other words, for each such material, that material (and only that material) is added as an additional node to that network (with the tie-lines it would form with materials existing in the network), and related network properties are then computed.

Temporal evolution of the networks can be traced in increments, for example, of 1, 2, 5, 10 years, between two consecutive times t_j. In the present work, we chose an increment of 5 years. These times form an m element long ordered tuple of times, T. For example $T = \{1940, 1945, 1950, 1955, 1960, 1965, 1970, 1975, 1980, 1985, 1990, 1995, 2000, 2005, 2010, 2015\}$ where $t_1 = 1940$, $t_2 = 1945$ etc.

A material i is included in the network when its discovery time t_d^i obeys $t_d^i \leq t_j$ for t_j in T. For example, if the proxy for the discovery time of a material is found to be $t_d^i = 1992$, in above example it will first appear as part of the network pertaining to 1995. Above processes yield m number of n element-long vectors $x_i(t_j)$ for a node i such as:

$$\mathbf{x}_i(t_1) = [k_i^1(t_1), k_i^2(t_1), k_i^3(t_1), ..., k_i^n(t_1)] \tag{1}$$

$$\mathbf{x}_i(t_2) = [k_i^1(t_2), k_i^2(t_2), k_i^3(t_2), ..., k_i^n(t_2)] \tag{2}$$

$$...$$

$$\mathbf{x}_i(t_{j=m}) = [k_i^1(t_m), k_i^2(t_m), k_i^3(t_m), ..., k_i^n(t_m)] \tag{3}$$

where $k_i^n(t_j)$ denotes the nth network property for material i at time t_j. For a material i, for times earlier than t_d^i, material would be given a label $y(t_j < t_d^i) = 0$ indicating that the discovery has not taken place, whereas for times later than or equal to t_d^i, material would be given a label $y(t_j \geq t_d^i) = 1$ indicating that the discovery has taken place. This process would yield an m-element long vector $\mathbf{Y}_i$ for material i. For the example above with

$t_d^i = 1992$ and time set T, one obtains $\mathbf{Y}_i = [0,0,0,0,0,0,0,0,0,0,0,1,1,1,1,1]$. Above processes yield, overall, a set of network property vectors corresponding to different times, $\mathbf{X}_i = [x_i(t_1), x_i(t_2), ..., x_i(t_{j=m})]$ for each material i and the corresponding target class labels $\mathbf{Y}_i$ for their status of discovery.

Model building for synthesizability: The $\mathbf{X}_i$ and $\mathbf{Y}_i$ derived with the procedure above can then be used to build models for estimation of likelihood of discovery, in other words, models -once trained with these inputs- that can be used to estimate likelihood of successful synthesis of computer-generated, hypothetical materials. In the present implementation, a sequential sliding window machine-learning method is employed to deconstruct the time-series formulation above to form a supervised learning problem. In this method, we extract sequences of a certain length (a window) from $\mathbf{X}_i$ by sliding the window one time step (j) at a time to obtain a sequence. Therefore feature vectors corresponding to times earlier than the time of interest (target time) is also included as part of the feature vector, depending on how wide w is. For example, for a window of size $w = 3$, the feature vector corresponding to target $y_i(t_5)$ for time t_5 includes a concatenated vector of $x_i(t_5)$, $x_i(t_4)$ and $x_i(t_3)$. The largest possible w is the number of time increments extracted, i.e. m, whereas the smallest possible w is 1. Past values of y_i can also be concatenated to the feature vector with a same size as or a different size, hence making the models "recurrent". For the present implementation, we found that recurrence was not required to obtain accurate results. The target class label is always the single value that corresponds to the latest time step included in the feature vector, and therefore it must not be included in the feature vector by accident. Given the feature vectors and corresponding class labels, any supervised machine-learning algorithm relevant for the problem can be employed, including but not limited to the logistic regression and random forest methods described in the manuscript. Models that provide well-calibrated probabilities in class predictions are preferred.

Predictive model application for synthesizability: Trained models can be applied to computer-generated (hypothetical) materials that are stable but not discovered, i.e. not synthesized and characterized experimentally, to predict their probability of successful synthesis today (which can be assumed to be well represented by the latest network studied in the time increments, e.g. 2015 in the present work). Model can also be interpreted as returning binary class labels synthesizable (1) vs unsynthesizable (0). Model can also be applied retrospectively to the hypothetical materials to predict when the collective circumstances

in the network were mature enough to enable their synthesis, returning a time increment where that material could have already been successfully synthesized. This time increment may further be interpreted as: the longer is the time that has passed since a hypothetical material was first predicted to be synthesizable, the higher the confidence would be in an attempt of synthesis would be successful.

SUPPLEMENTARY NOTE 1

Below we list the hypothetical compounds in the OQMD that are predicted as synthesizable with 95% probability with the network-based synthesizability method. Whether a stable compound was experimentally-known or not was decided automatically by checking an entry against the ICSD, which serves as our assumed ground-truth for experimental structures. Therefore, for various reasons such as the lack of a corresponding ICSD entry, having partially-occupied sites in the crystal structures, or having an erroneous entry, there is always the possibility that an experimental structure may be missing from the OQMD, and therefore a hypothetical structure may be listed below as synthesizable. With alphabetical order: Ac_2CuGe, Ac_2CuIr, Ac_2CuSi, Ac_2CuSn, Ac_2GaCu, $AcAgO_3$, $AcAlO_3$, $AcAu_3$, $AcAuO_3$, $AcCoO_3$, $AcCrO_3$, AcCu, $AcCuO_3$, $AcErO_3$, $AcFeO_3$, $AcGaO_3$, $AcInO_3$, $AcLuO_3$, $AcMnO_3$, $AcNiO_3$, $AcPdO_3$, $AcScO_3$, $AcSe_3$, $AcTlO_3$, $AcTmO_3$, Ag_2Cl_3, Ag_2F_3, AgAu, $AgAu_3$, $AlAg_3$, BIr, BRu, $Ba_2Ga_2O_5$, $Ba_2Sc_2O_5$, $BaCe_2O_4$, $BaDy_2O_4$, $BaLa_2O_4$, BaNa, Be_2CuIr, Be_2CuPt, Be_2CuRh, Be_2CuRu, Be_2RuPt, Be_3Ir, Be_3Rh, Be_3Ru, BiP, $CaHfO_3$, $CaPdO_3$, Cd_3Rh, $CdAg_3$, $CdPd_3$, $CdPdO_3$, $CdPtO_3$, $CeAgO_3$, $CeAu_3$, $CeAuO_3$, $CeCoO_3$, $CeFeO_3$, $CeGa_3$, $CeGaO_3$, CeI_3, CeIn, $CeInO_3$, $CeMnO_3$, $CeNiO_3$, $CePdO_3$, $CeZn_2Ag$, Cs_2F_3, $CsSn_3$, $CuPt_3$, Dy_2Mg, $DyAlO_3$, DyF_3, $ErAlO_3$, $ErCl_3$, $ErMg_3$, $Eu_2Ga_2O_5$, Eu_2N, Eu_3P, Eu_3Sn, $EuAgO_3$, $EuAuO_3$, $EuBeO_3$, $EuBiO_3$, $EuCoO_3$, $EuGeO_3$, $EuHfO_3$, $EuIn_2O_4$, $EuNpO_3$, $EuPaO_3$, $EuPbO_3$, $EuPuO_3$, $EuSc_2O_4$, $EuSnO_3$, $EuTm_2O_4$, Fe_2CuO_4, $GaCu_3$, $GaCuRh_2$, $GaNi_2O_4$, $GaSn_3$, Gd_2Co_{17}, Gd_2Mg, GdF_3, GdI_3, GdIr, Hf_2CuIr, Hf_2CuOs, Hf_2CuTc, $HfCdCu_2$, $HfCdO_3$, HfFe, $HfZn_3$, $HgPd_3$, $HgPdO_3$, $HoCl_3$, $HoMg_3$, HoRu, In_2HgO_4, $InCl_3$, IrO_3, $KAsO_3$, $KBiO_3$, $KPaO_3$, KSn_3, $LaAgO_3$, $LaAu_3$, LaH_3, Li_2CoO_3, Li_2FeO_3, Li_2OsO_3, Li_2PdO_3, Li_2TcO_3, Li_2ZnCu, Li_3Ag, $Li_3Ce_3Se_2O_{12}$, $Li_3Gd_3Se_2O_{12}$, $Li_3La_3Se_2O_{12}$, $Li_3Nd_3Se_2O_{12}$, $Li_3Os_2O_6$, Li_3Pt, $Li_3Sm_3Se_2O_{12}$, $Li_3Tc_2O_6$, $Li_3Y_3Se_2O_{12}$, $Li_3Yb_3Se_2O_{12}$, $Li_3Yb_3Te_2O_{12}$, Li_5CoO_4, Li_5Mg, Li_6MnO_4, LiAu,

$LiBe_2Ir$, $LiCu_3$, $LiGa_3$, $LiGeAu_2$, $LiIn_3$, $LiIr_2O_6$, $LiIrO_3$, LiMg, $LiMg_2$, LiNi, $LiOsO_3$, $LiPaRu_2$, $LiPd_3$, $LiPm_2Al$, $LiPm_2Ga$, $LiPm_2Ir$, $LiPm_2Pt$, $LiPm_2Rh$, $LiPm_2Ru$, $LiPt_3$, $LiTcO_3$, $LiZn_3$, $LiZn_5$, $LuAlO_3$, $LuCl_3$, LuCu, $LuMg_3$, $LuSn_3$, Mg_3Ag, Mg_3Tl, $MgPd_5$, MgPt, $MgRh_3$, $MgSn_3$, $MgSn_5$, Mn_2CrCo, Mn_2S_3, MnH, MnV_3, Na_2MnO_3, Na_2TiO_3, Na_2ZrO_3, Na_3Li, Na_3Pd, Na_3Tl, Na_3VO_4, $Na_4Ge_9O_{20}$, NaB_3O_5, $NaIn_3$, $NaMoO_3$, $NaPaO_3$, NaPd, $NaRuO_3$, $NaScO_2$, $NaTl_3$, $NaYO_2$, Nb_3Ru, $NdAu_3$, NdIn, $NiPbO_3$, Np_2O, Np_2O_3, Pa_3Ga, Pa_3Sn, $PaAgO_3$, $PaAu_3$, $PaAuO_3$, $PaCo_3$, $PaCu_2Sn$, $PaCuO_3$, $PaGaCu_2$, $PaHgO_3$, $PaInCu_2$, PaO_3, $PaPt_3$, $PaSe_3$, $PaSi_3$, PdO_2, Pm, Pm_2AgRu, Pm_2CoIr, Pm_2CuGe, Pm_2CuIr, Pm_2CuPt, Pm_2CuRh, Pm_2CuRu, Pm_2CuSi, Pm_2IrRu, Pm_2NiRu, Pm_2PdRu, Pm_2RuAu, Pm_2RuRh, Pm_2ZnRu, $PmAgHg_2$, $PmAlCu_2$, $PmAlO_3$, $PmAu_3$, $PmCoO_3$, $PmCrO_3$, $PmCu_2Sn$, $PmCuO_3$, PmF_3, $PmFeO_3$, $PmGaCu_2$, $PmGaO_3$, $PmGeAu_2$, PmH_3, $PmInCu_2$, PmIr, $PmMnO_3$, $PmNiO_3$, $PmPd_3$, $PmPdO_3$, $PmPt_3$, PmRu, $PmScO_3$, PmSe, $PmSn_3$, $PrAgO_3$, $PrAuO_3$, $PrCuO_3$, PrI_3, Pu_3O_2, $RbAsO_3$, RbF_2, $RbMnO_3$, RbNa, $RbSn_3$, $ReRu_3$, Sc_2CdO_4, ScIn, $ScZn_3$, $SeBr_2$, SeI_2, SiTc, $SmAl_3$, $SmCl_3$, SmIr, SnGe, $SrPdO_3$, SrPt, $SrPuO_3$, SrY_2O_4, Ta_3Ru, $TaCuRh_2$, TaMn, TaP, $TeBr_2$, $ThAu_3$, $ThPt_3$, Ti_3Tc, TiMn, TiN, TiPt, Tl_2HgO_4, Tl_3Te, TmPt, TmRu, U_3Ge, UFe, UP_3, VFe_3, WN_2, Y_2Mg, YMg_3, $YbCd_3$, YbF_2, $YbGeO_3$, $YbHfO_3$, $YbIrO_3$, YbN_2, YbO_2, YbP, $YbPbO_3$, $YbPdO_3$, $YbPtO_3$, $YbSnO_3$, $YbTeO_3$, $YbZn_5$, $YbZrO_3$, Zn_2CuRh, Zn_3Cu, Zn_3Ir, Zn_3Os, Zn_3Pd, Zn_3Pt, Zn_3Rh, $ZnCu_3$, $ZnCuPd_2$, ZrSe are $ZrZn_3$ are predicted to have 95% or more chance of being made in the laboratory if attempted today.

SUPPLEMENTARY FIGURES 1 TO 11

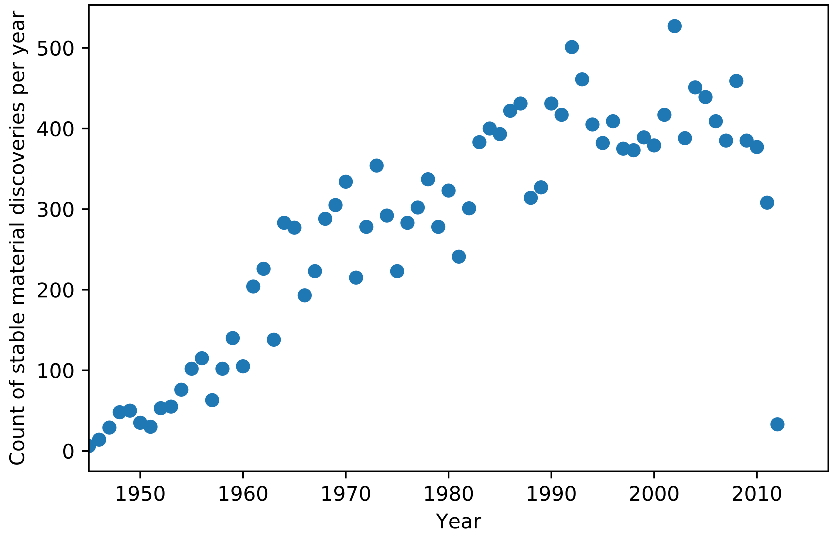

Supplementary Figure 1. **Number of stable materials discovered as a function of time.** While the pace of discovery has been increasing, number of discoveries per year on and after 1960s have the same order of magnitude (few hundreds), ensuring discoveries are not too concentrated in one time but are more spread out in the data set.

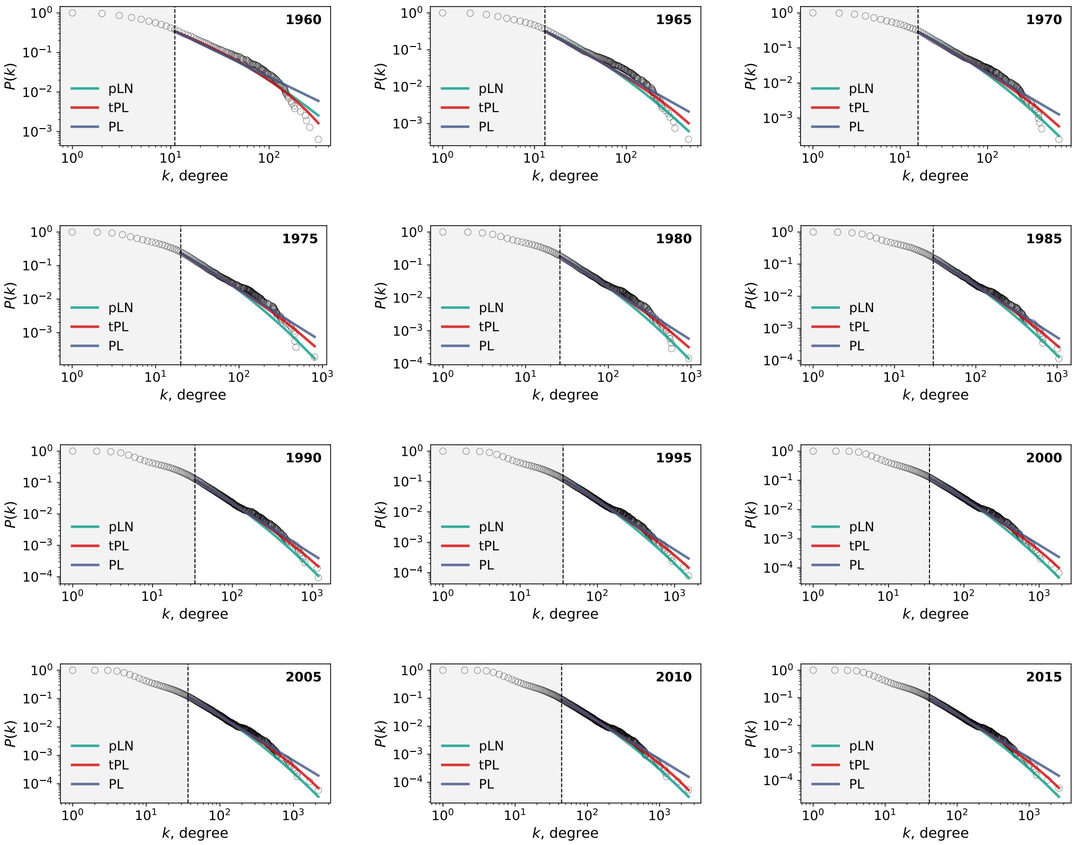

Supplementary Figure 2. **Degree distributions of stability networks.** Power-law, truncated power-law and positive log-normal distributions are denoted as PL, tPL and pLN, respectively.

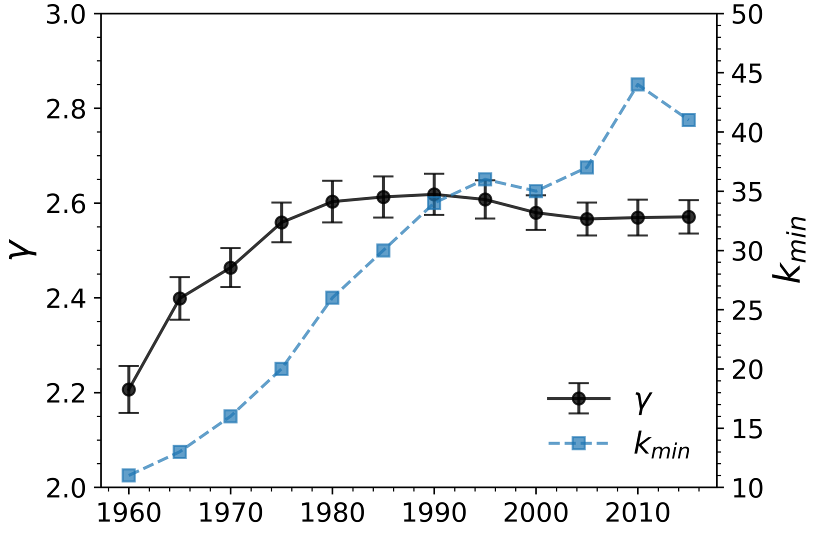

Supplementary Figure 3. **Fitted distribution parameters.** Time evolution of exponent γ and k_{min} for fitted power-laws for degree-distributions in material stability networks - the subset of full convex-hull network relevant for material stability.

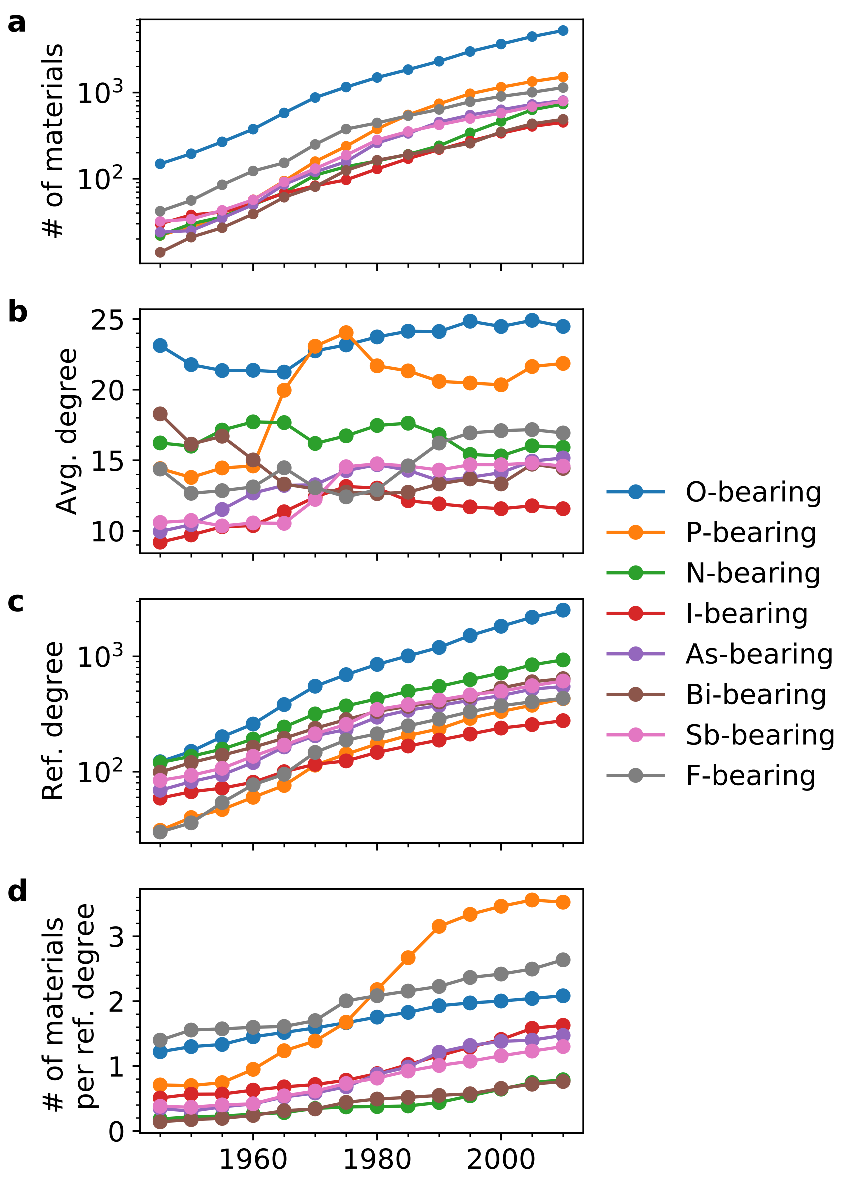

Supplementary Figure 4. Evolution of the network size and degrees in different chemistries. Evolution of number of materials in respective chemistries shown in the legend is shown in (a) and the evolution of the average degree of such materials are shown in (b). The degree of the reference elements that broadly define the "chemistry", which intuitively serve as major hubs in their respective chemistries, are shown in (c). The chemistries noted as O-bearing or P-bearing, and so on, are defined to encompass all materials that contain some amount of the that reference element, e.g. oxygen and phosphorus in these examples. The number of materials in a given "chemistry" per degree of those reference elements defining those chemistries are shown in (d).

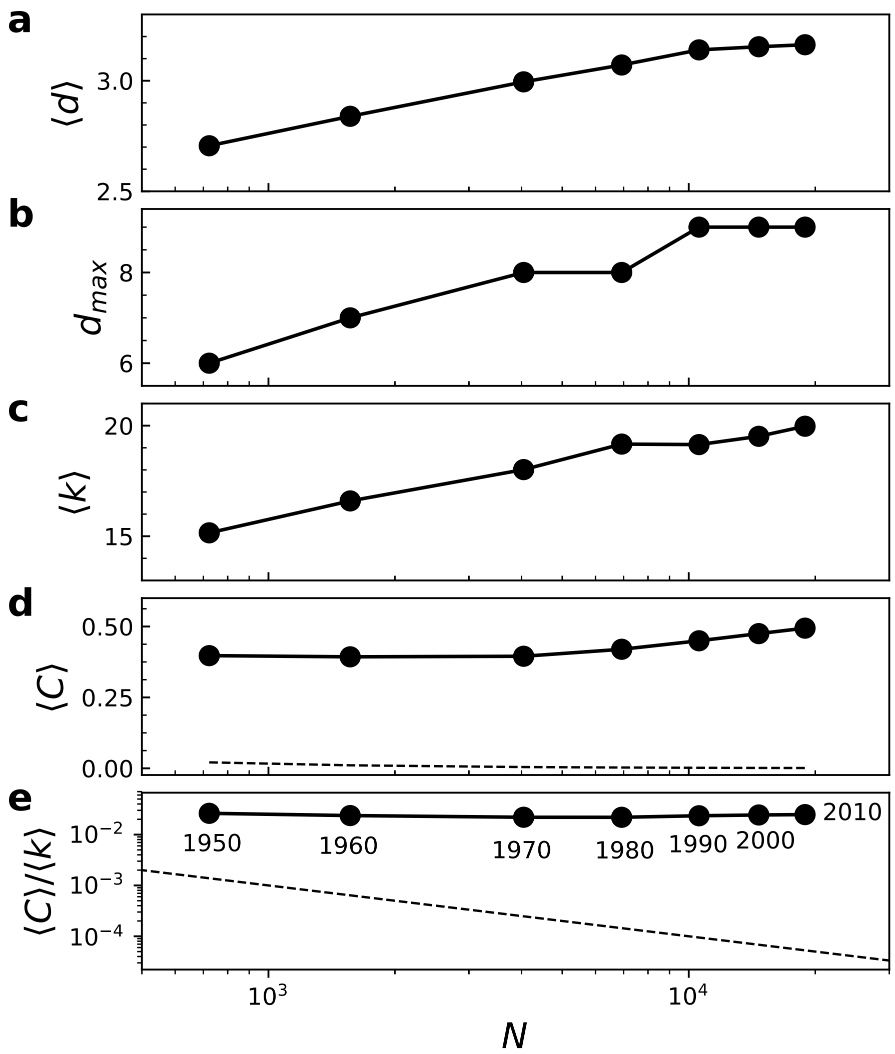

Supplementary Figure 5. **Evolution of metrics of the materials stability network.** Changes in (a) average path length $\langle d \rangle$, (b) diameter d_{max} (longest shortest-path length), (c) average degree $\langle k \rangle$, (d) average clustering coefficient $\langle C \rangle$ and (e) ratio of $\langle C \rangle$ to $\langle k \rangle$ with size of the network, N. Dashed lines represent expected values for the corresponding random networks. These global averages of properties of the network demonstrate its steady evolution. The recent average path length of 3.2 is relatively small compared to the typical scale-free networks. The diameter has reached slowly to 9 and leveled out. As expected from densification, the average degree is increasing and has reached ∼20. The nodes in real networks tend to cluster compared to random graphs, which also is the case for the materials stability network as early as in 1950s.

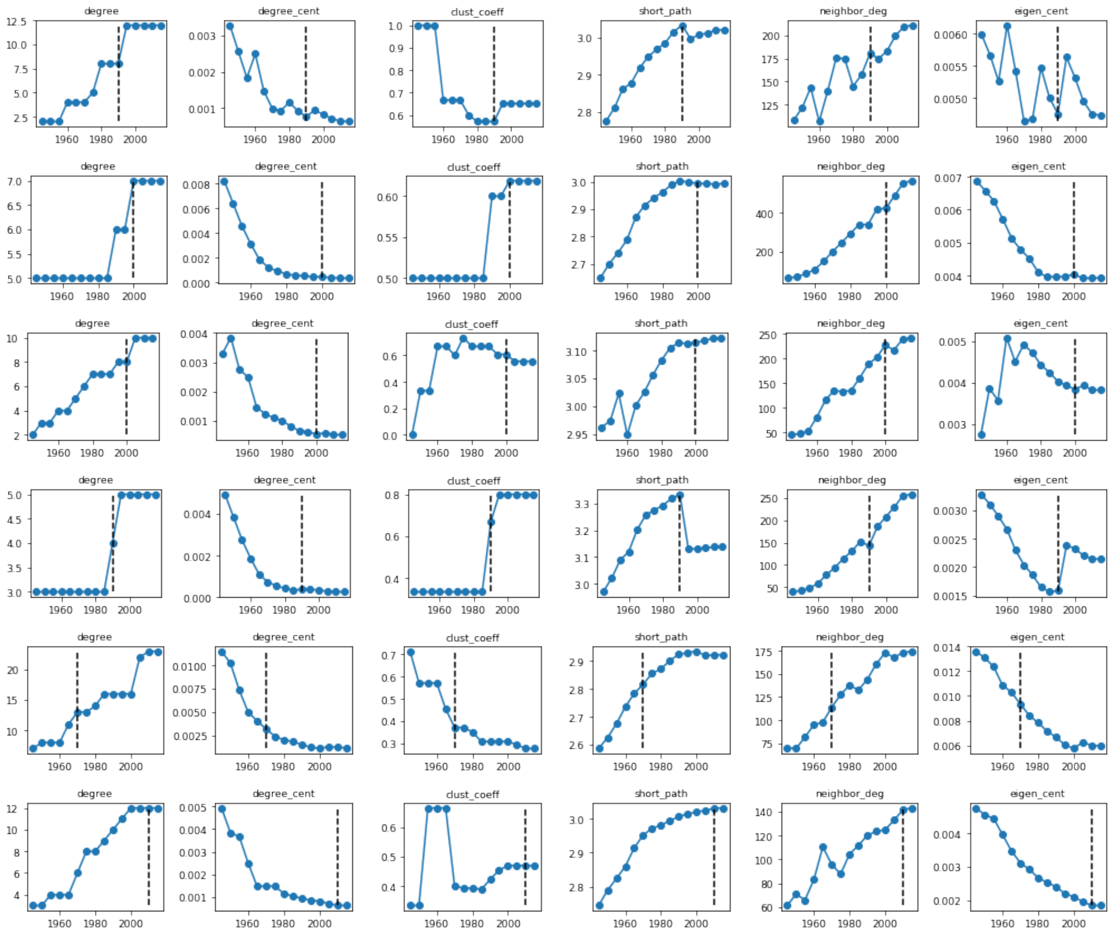

Supplementary Figure 6. **Evolution of network properties for six sample materials.** These materials are $Li_5CsB_2O_5$, $GdPd_2O_4$, Ba_2CoIrO_6, $BaTb_2PdO_5$, $DyZn_{12}$ and $Yb_3Ni_7B_2$ (top to bottom). Materials are chosen randomly. Dashed lines show the publication-extracted discovery time of the materials. Network properties include degree (degree), degree centrality (degree_cent), clustering coefficient (clust_coeff), mean shortest path (short_path), degree of neighbors (neighbor_deg) and eigenvector centrality (eigen_cent), labels in parentheses.

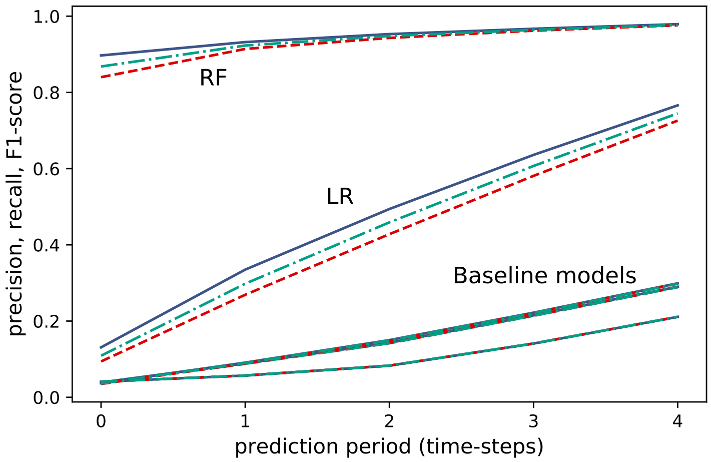

Supplementary Figure 7. **Event detection performance metrics as a function of the length of the prediction period.** LR and RF refer to the logistic regression and random forest models, respectively. Baseline models are class distribution prediction, constant positive class prediction and random choice, respectively. Baseline models are not labeled separately for clarity, as they follow similar trends. Fore each model, solid, dashed, and dot-dashed lines correspond to precision, recall and F1-score, respectively.

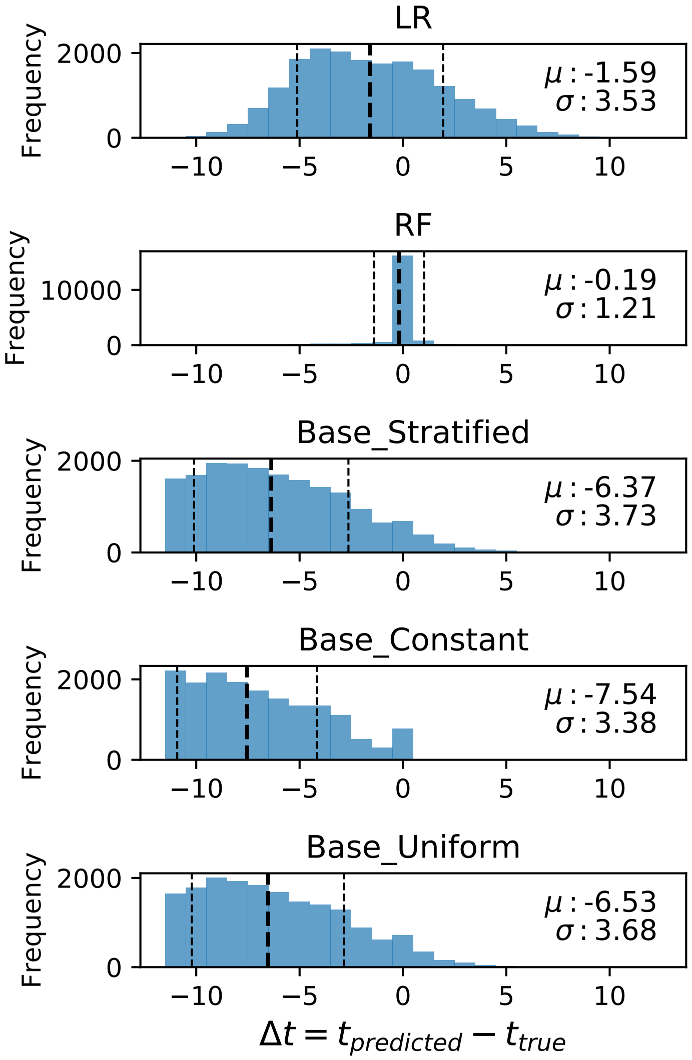

Supplementary Figure 8. **Distributions of model prediction errors relative to true discovery times of materials.** LR and RF refer to the logistic regression and random forest models, respectively. Baseline models are labeled as Base_Stratified, Base_Most-frequent and Base_Uniform, referring to class distribution prediction, positive class prediction and random choice, respectively. Errors were determined using 5-fold cross-validation over all materials. Bold and thin dashed-lines show locations of mean (μ) and standard deviation (σ), respectively.

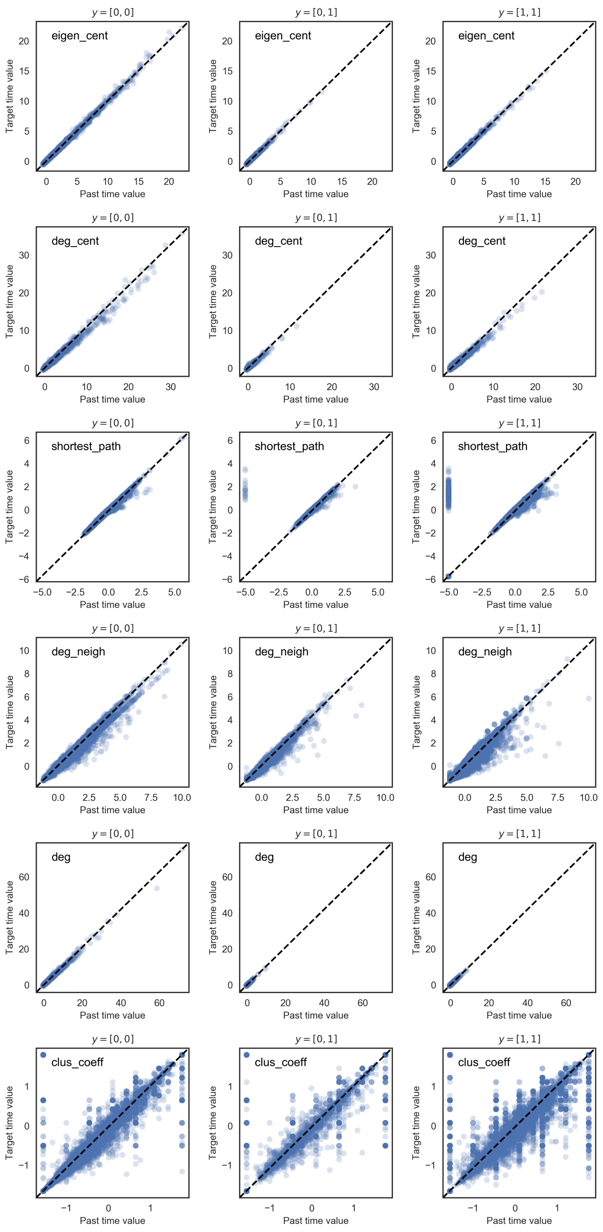

Supplementary Figure 9. Pair-wise comparisons of past time (pt) and target time (tt) values of the network properties of materials for subclasses $y = [0, 0]$ (no discovery yet at tt), $y = [0, 1]$ (just discovered at tt) and $y = [1, 1]$ (already discovered). We observe that pt and tt values reflect variations in time for most of the properties. Labels are deg for degree, deg_cent for degree centrality, shortest_path for mean shortest path, deg_neigh for degree of neighbors and clus_coeff for clustering coefficient.

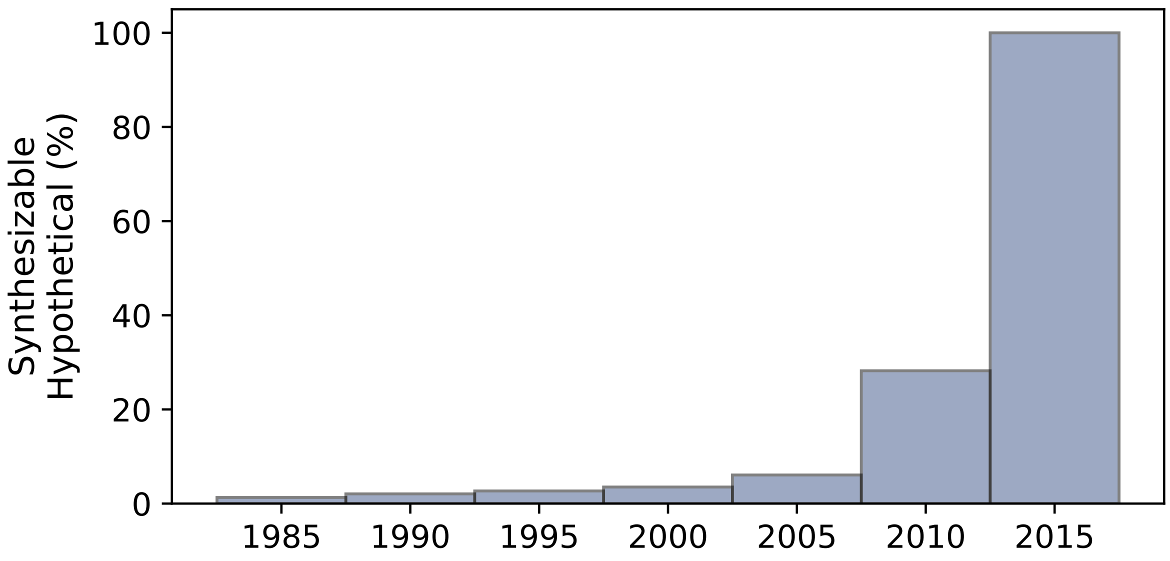

Supplementary Figure 10. Cumulative distribution of past-synthesizability of hypothetical materials predicted to be synthesizable today (based on the RF model) within the last few decades. This estimation is performed by applying the model to network-property based, past-time vectors (sequences) of hypothetical materials.

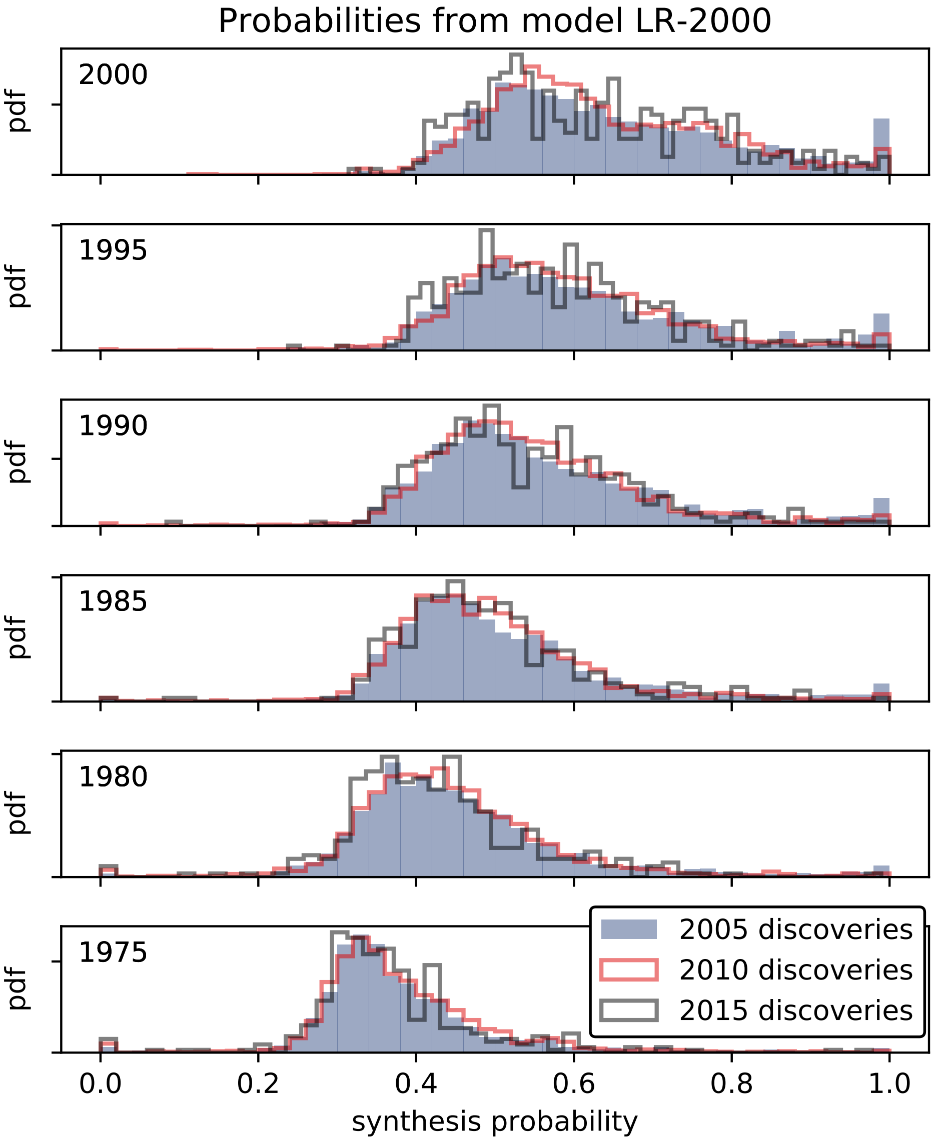

Supplementary Figure 11. **Probability distributions of future discoveries from an LR model trained with data until year 2000.** Future discoveries include materials known to be discovered in periods through 2005, 2010, and 2015. LR probabilities were further calibrated using the isotonic regression.

SUPPLEMENTARY TABLES 1 TO 3

Supplementary Table 1. Comparison of goodness of fit of several viable distributions to a power law distribution. R and p-value are listed in the format [R, p] in each cell. R is the log-likelihood ratio between a particular distribution and corresponding power-law. p-values are calculated using Kolmogorov-Smirnov statistics and bootstrapping.(See Ref. 36 in the manuscript).

Year	Exponential	Lognormal	Positive lognormal (pLN)	Power law (PL)	Stretched exponential	Truncated power_law (tPL)
1960	[98.4808884792, 5.39899417156e-09]	[-2.96674198137, 0.0994851048324]	[-2.13604529045, 0.452161856419]	[0.0, 1.0]	[-3.74956775667, 0.0904845779186]	[-7.84703913283, 7.44569158928e-05]
1965	[244.996581014, 1.14584048188e-21]	[-0.00555738237012, 0.93819254176]	[10.6200957888, 0.00373873186154]	[0.0, 1.0]	[3.02853069694, 0.155636165056]	[-2.10075650922, 0.0403879291218]
1970	[306.472076383, 4.15946418333e-22]	[-0.0294477823205, 0.862546121317]	[11.0839087852, 0.00494842882323]	[0.0, 1.0]	[4.11101650226, 0.110083770956]	[-1.94292747848, 0.0486944639865]
1975	[324.83419697, 2.4137569217e-21]	[0.00823340542718, 0.859347947974]	[12.1738942146, 0.0020171806814]	[0.0, 1.0]	[7.67472495176, 0.0153291884548]	[-1.15905225372, 0.127875766472]
1980	[293.891764488, 4.92457478761e-20]	[0.0122883311274, 0.839585367947]	[10.2055901416, 0.00407099752372]	[0.0, 1.0]	[4.76373622455, 0.0491062489367]	[-1.02359125271, 0.152488157678]
1985	[298.175105311, 3.16951597089e-17]	[-0.0277989269893, 0.873684333138]	[8.1219255608, 0.0228859269077]	[0.0, 1.0]	[4.66599776218, 0.0929213392065]	[-1.11424831234, 0.135485619701]
1990	[300.008260452, 5.72381967756e-17]	[-0.0340851794404, 0.861354096712]	[7.64574153909, 0.0293682465095]	[0.0, 1.0]	[3.97245601751, 0.129177649477]	[-1.10296179432, 0.137481531518]
1995	[353.659613289, 4.17214384021e-18]	[-0.0216506092872, 0.890298002025]	[8.97944467685, 0.0175420444261]	[0.0, 1.0]	[5.14482013791, 0.0827953482975]	[-1.16574011652, 0.12678126831]
2000	[427.984459949, 6.05218609624e-19]	[-0.133416369808, 0.738958475856]	[9.3018678839, 0.0273469179911]	[0.0, 1.0]	[4.66482330126, 0.140280962884]	[-1.47663399779, 0.0857040893313]
2005	[468.938914914, 7.17372454261e-19]	[-0.262665772509, 0.645565872781]	[9.01067301665, 0.0408974182474]	[0.0, 1.0]	[5.30689733264, 0.143432795814]	[-1.79326587672, 0.0582496926573]
2010	[389.727629264, 2.98748117108e-16]	[-0.306150444042, 0.614388016224]	[6.55818041798, 0.0878495568268]	[0.0, 1.0]	[3.19275418523, 0.285526806685]	[-1.74250391798, 0.0619270681723]
2015	[461.480589121, 5.21872638166e-18]	[-0.144723971519, 0.731361356816]	[9.25715708498, 0.029836675687]	[0.0, 1.0]	[5.48215521599, 0.109340129571]	[-1.53224528412, 0.0800202408279]

Supplementary Table 2. Parameters of power-law (PL) and truncated power-law (tPL) degree distributions fitted to material stability network. Symbols γ, σ and β correspond to the power exponent of the power-law, standard deviation, and exponential factor in the tPL (i.e. PL with an exponential cutoff.)

Year	γ PL	σ	k_{min}	γ tPL	β tPL
1960	2.206505	0.049462	11	1.904162	0.00435
1965	2.398539	0.044766	13	2.296578	0.00126
1970	2.463631	0.041233	16	2.374899	0.00095
1975	2.55883	0.041962	20	2.491926	0.00062
1980	2.602855	0.0441	26	2.53506	0.00051
1985	2.612748	0.043524	30	2.541679	0.000472
1990	2.618192	0.043435	34	2.547506	0.000416
1995	2.607336	0.04036	36	2.541584	0.000358
2000	2.579527	0.036615	35	2.513105	0.000361
2005	2.566278	0.034721	37	2.496203	0.000357
2010	2.569173	0.037596	44	2.492185	0.000335
2015	2.570769	0.035381	41	2.506195	0.000295

Supplementary Table 3. Estimated synthesis probabilities of recent computational discoveries for diverse applications.

**Layered Li_2ABO_6**	**Heusler alloys**		**Perovskites**
Li_4CoTeO_6 0.73	$TiMn_2Al$ 0.63	$MnSnRu_2$ 0.92	$PuGaO_3$ 0.54
Li_4MnTeO_6 0.65	$TiMn_2Ga$ 0.79	$MnSbRu_2$ 0.91	$CaPuO_3$ 0.58
Li_4NiTeO_6 0.85	$TiMn_2Si$ 0.70	$FeGeRu_2$ 0.94	$EuCrO_3$ 0.58
Li_4ZnTeO_6 0.69	$TiMn_2Ge$ 0.70	$ScAlCo_2$ 0.64	$EuIrO_3$ 0.58
Li_4AlIrO_6 0.72	Mn_2VSi 0.67	$ScGaCo_2$ 0.66	$EuRuO_3$ 0.62
Li_4AlOsO_6 0.71	Mn_2VGe 0.80	$TiInCo_2$ 0.56	$SrPaO_3$ 0.65
Li_4AlPtO_6 0.65	Mn_2AlCr 0.91	VCo_2Ge 0.63	$EuNbO_3$ 0.70
Li_4AlRhO_6 0.62	Mn_2FeSi 0.82	$CrCo_2Ge$ 0.78	$EuMoO_3$ 0.72
Li_4AlRuO_6 0.79	Mn_2FeGe 0.85	$ScAlRh_2$ 0.58	$SrNpO_3$ 0.75
Li_4AlSbO_6 0.74	$TiFe_2Si$ 0.68	$ScGaRh_2$ 0.76	KWO_3 0.79
Li_4CoBiO_6 0.66	$TiFe_2Ge$ 0.82	$ScInRh_2$ 0.57	$EuVO_3$ 0.84
Li_4CoSbO_6 0.73	Fe_2CoSi 0.70	$ScSnRh_2$ 0.74	$YbRuO_3$ 0.84
Li_4CrBiO_6 0.66	Fe_2CoGe 0.72	$ScSbRh_2$ 0.59	$SrUO_3$ 0.9
Li_4CrSbO_6 0.63	$ScGaRu_2$ 0.60	$TiAlRh_2$ 0.66	$CsPaO_3$ 0.91
Li_4CuOsO_6 0.79	$ScSnRu_2$ 0.58	$TiGaRh_2$ 0.79	$CeCuO_3$ 0.91
Li_4DyOsO_6 0.73	$ScSbRu_2$ 0.57	$TiInRh_2$ 0.71	$CeScO_3$ 0.91
Li_4ErOsO_6 0.72	$TiAlRu_2$ 0.59	$TiSnRh_2$ 0.73	$EuTiO_3$ 0.91
Li_4FeSbO_6 0.82	$TiGaRu_2$ 0.60	$CrGaRh_2$ 0.57	$TbCuO_3$ 0.92
Li_4GaOsO_6 0.71	$TiSiRu_2$ 0.60	$MnGaRh_2$ 0.74	$YbRhO_3$ 0.93
Li_4GaRuO_6 0.69	$TiGeRu_2$ 0.63	$MnInRh_2$ 0.74	$PrCuO_3$ 0.95
Li_4GaIrO_6 0.67	$TiSnRu_2$ 0.60	$MnSiRh_2$ 0.66	$EuCoO_3$ 0.97
Li_4GdOsO_6 0.64	$AlVRu_2$ 0.57	$AlFeRh_2$ 0.75	$EuPbO_3$ 0.97
Li_4HoOsO_6 0.72	$VGaRu_2$ 0.56	$GaFeRh_2$ 0.75	$EuHfO_3$ 0.98
Li_4InBiO_6 0.70	$VSiRu_2$ 0.58	$GaNiRh_2$ 0.79	$EuPaO_3$ 0.98
Li_4InOsO_6 0.72	$VGeRu_2$ 0.60	Mn_2FeSi 0.82	$EuPuO_3$ 0.98
Li_4InSbO_6 0.65	$AlCrRu_2$ 0.82	Mn_2FeGe 0.85	$EuSnO_3$ 0.98
Li_4MnSbO_6 0.65	$CrGaRu_2$ 0.71	Fe_2CoSi 0.70	$CeInO_3$ 0.99
Li_4NiSbO_6 0.63	$CrSiRu_2$ 0.71	Fe_2CoGe 0.72	$CeCoO_3$ 1.0
Li_4SbRhO_6 0.52	$CrGeRu_2$ 0.72		$CeNiO_3$ 1.0
Li_4ScOsO_6 0.73	$MnAlRu_2$ 0.91		$EuGeO_3$ 1.0
Li_4TbOsO_6 0.73	$MnGaRu_2$ 0.83		
Li_4TlOsO_6 0.63	$MnSiRu_2$ 0.84		
Li_4YOsO_6 0.74	$MnGeRu_2$ 0.84		
