# Supplementary material for: Network analysis of synthesizable materials discovery
Source: Nat Commun. 2019 May 1;10:2018. doi: 10.1038/s41467-019-10030-5 (PMC6494829; doi:10.1038/s41467-019-10030-5)
Supplement: Supplementary file 2 — Description of Additional Supplementary Files [file 41467_2019_10030_MOESM2_ESM.pdf]

Description of Additional Supplementary Files

File Name: Supplementary Data 1
Description: Materials stability network and its temporal properties.
